# Creating a dataset of historic roads in Sydney from scanned maps

**DOI:** 10.1038/s41597-023-02574-5

**Published:** 2023-10-07

**Authors:** Hamish Turner, Bahman Lahoorpoor, David M. Levinson

**Affiliations:** School of Civil Engineering, University of Sydney, Sydney, Australia

**Keywords:** Civil engineering, Geography

## Abstract

This study creates a historic dataset of road opening dates in Sydney. A method was developed for map digitization to extract spatial data from historic maps and place them in a collective vector layer. The method includes extensive georeferencing of the maps, as well as editing and cleaning the maps through raster and vector analysis. Preferred methods for map digitization used in the project were identified. For a considerable area of Sydney, in which approximately 52000 road links were included, almost half of the links were identified with an open date by the start of the twentieth century. A further half of these links were confined to opening within a thirty-year period. The project has established a strong foundation for a historic road dataset for Sydney. It has also outlined methods and procedures that can be followed to progress the dataset further.

## Background & Summary

For historians, engineers, planners, researchers, and other interested parties, there is value to be gained from understanding the evolution of road networks. Historical records can serve to benefit cities moving forward at the strategic, tactical, and practical levels. Examples are abundant. For instance, the environmental impacts of road development and subsequent land impact can be assessed over time, while roads in need of reconstruction, conservation, or modification can be identified from their construction dates. For example, Cardew *et al*. investigated the recent construction of large residential areas in Sydney’s West^1^. Certain roads were identified as necessities to support developing manufacturing industries. Similarly, Walsh focuses on the relevance of geography to progressing industries in the early colonial period of Sydney’s history^2^.

Studying the evolution of road networks requires digitizing historic records and maps. Map digitizing is a type of spatial modeling that integrates image processing and automation techniques. The development of these techniques to provide data into a GIS environment has been the focus of research since the 1990s^3–6^. Thanks to these efforts, today’s road network is accessible from various sources such as OpenStreetMap (OSM). However, OSM generally lacks historic information about each segment (i.e., road opening and closure dates) and little effort has been put into extracting this information from historic records and maps.

Recent literature puts significance on the digitized geography of historic sources. Historic GIS, which integrates the study of historic sources such as artifacts, data and maps with Geographic Information Systems (GIS), adds new dimensions to the base of historic information^7^. In summary, GIS is a representation of qualitative and quantitative data through attributes and spatial characteristics, allowing spatially defined geographic data to be used to enhance historic inquiries^8^.

Over time, GIS has developed into a tool useful for expressing urban development. Loren Siebert has developed a GIS dataset for understanding the urbanization of Tokyo since the late nineteenth century^9^. The source creates a spatial history by integrating numerous historic sources^10^. This includes physically tracing landscapes as a vector, uploading census data as attributes and utilizing rail construction detail, as a means of tracking demographic change over time^7^.

One of the first major historic GIS datasets developed was the Great Britain Historical GIS Project (GBHGIS) to trace the changing administrative boundaries of previous data the project’s historians had collected^11^. Administrative boundaries were also traced in another earlier project on the history of China (222 BCE - 1911 AD)^12^. The two works were formative for the historic GIS field and explored various ways of using GIS tools to represent historic data.

Several historic GIS projects have considered road development in their research. A project aimed at reconstructing Rome from the eighteenth century uses a base map from 1748 by Giovanni Battista Nolli^13^. The map is digitized and georeferenced so that it can be compared to vector GIS layers. Overlays of vector layers, such as pedestrian paths and polygons of structures, against the georeferenced base map are used to assess the maps accuracy. In a separate project, Levin *et al*. from the Hebrew University of Jerusalem have used GIS as a tool to extrapolate roads from digitized and geoprocessed maps^14^. Similarly, Perret *et al*. have developed some methods to digitize French road network at national level by the historic maps of Cassini in the 18th century^15^.

Other studies investigated the evolution of road network growth, the topological characteristics and urban morphology of different case studies. For example historic road networks in London^16^, Paris^17^, Zurich^18^, Changchun^19^, Seoul^20^, Milan^21^, Minneapolis^22^ and other cities^23–25^ have been digitized and processed. Hypothetical (synthetic) networks were also explored^26–28^.

Another notable project comes from academic Andrew Wilson, who has used GIS to generate a historic dataset of Sydney^29^. Since 1998, the project has digitized historic maps, images, verbal descriptions and other sources to form the basis of its dataset. However, the work has rarely considered the geographic significance of road construction in Sydney. Roads have been viewed as a means of connecting points in history worth discussing and the importance of their location in space has not been considered the center of discussion itself. This study aims to provide a dataset so that the location of roads in both Sydney’s space and time can be properly considered.

Other projects with similar ambitions to create a historic road dataset have chosen to manually complete the map digitization process, usually by simply drawing vectors by tracing a historic map. The New York Public Library’s *Spacetime* project has crowd-sourced users to complete this tracing with voluntary input^30^. Another project focusing on the change in road structure in Manila over a hundred-year period concluded that it was more efficient to trace lines manually after an automated georeference transition was completed^31^.

Historical maps are usually in the form of scanned maps, which need raster classification for processing. There are many supervised and unsupervised methods described in the literature. A common raster classification technique is called color image segmentation (CIS). Color image segmentation is the process of dividing an image into multiple segments or regions based on color attributes using different clustering algorithms^32–37^. In a raster classification, the goal of this technique is to separate objects or regions with similar color values and assign them a unique label. It contributes to the creation of a clearer and more concise representation of the image, making it easier to analyze and process further.

Furthermore, there are a few state-of-the-art studies using machine learning methods for map digitization. For example, text detection in historic maps^38^, segmentation and digitization of historic maps^39,40^, feature recognition^41,42^, road extraction^43^, detecting road types^44^, and cadastral boundary extraction^45,46^ using neural network, deep neural network, and convolutional neural network models.

**Fig. 1:**
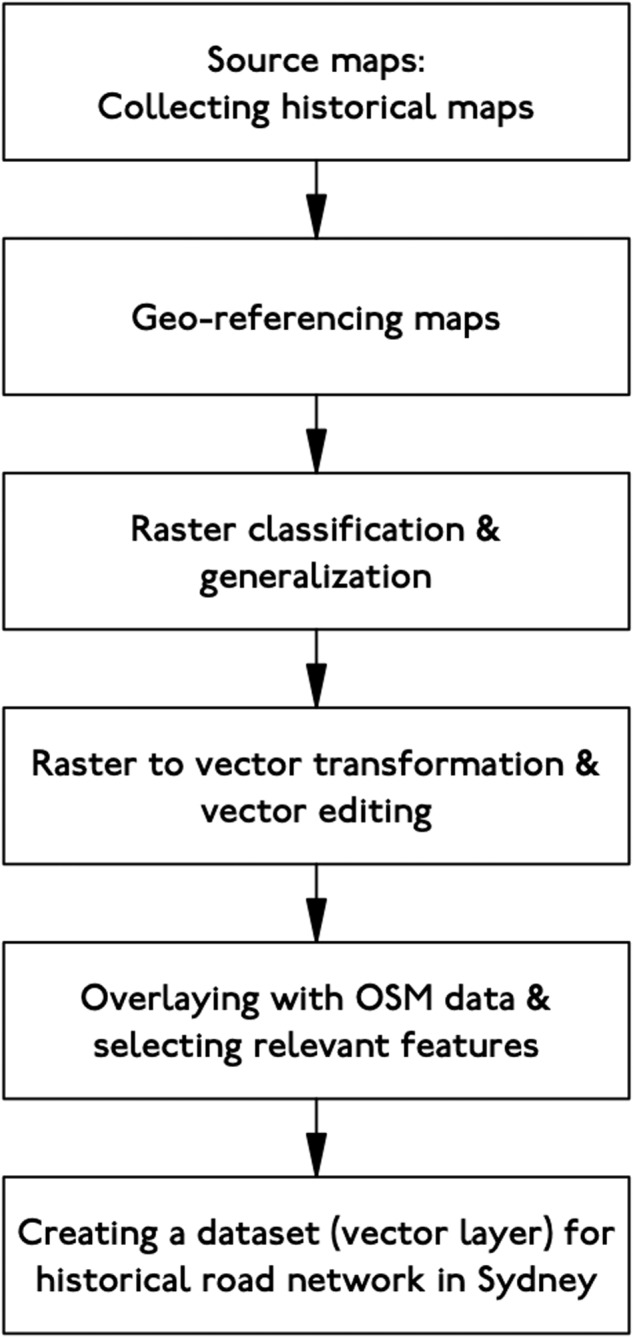
Conceptual framework of the study.

This study aims to digitize historic records for roads in Sydney, detailing the open and closure dates for each road link in the Sydney network. The project will collate the scattered historic records currently available for the Sydney road network into an accessible dataset, observing and identifying change over time. We aim to identify an efficient means of automating this process. Figure 1 provides a schematic overview of the study.

## Methods

The following subsections describe collecting data and the typical steps for map digitization. For all spatial processing and analysis in this study, QGIS version 3.22.15 (LTR) was used.

### Source maps

**Fig. 2:**
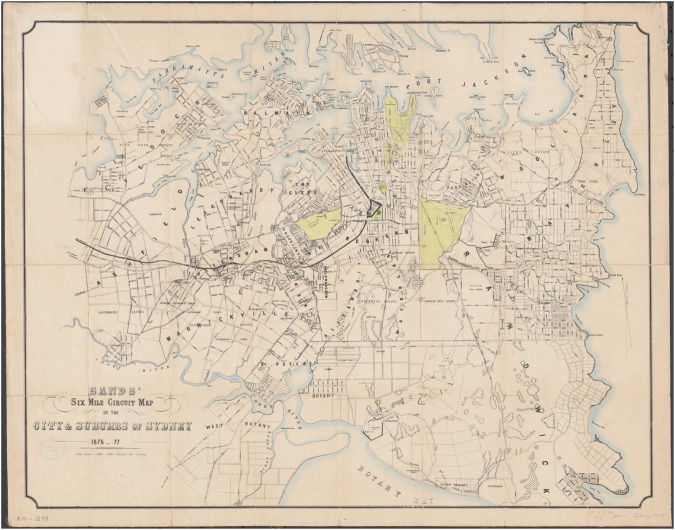
John Sands Map of the City and Suburbs of Sydney, 1877^63^.

There are volumes of historic maps available on the road networks of Sydney. Eight maps were selected, including maps from the National Library of Australia’s Trove database, published in 1877^47^ and 1903^48^, from the State Library of NSW for the map, published in 1890^49^, from the City of Sydney and *Dictionary of Sydney* for the maps published in 1844^50^, 1855^51^, 1888^52^, 1894 and 1886^53^. All of these maps are out of copyright and publicly available online in image format. The John Sands Map of the City and Suburbs of Sydney, published in 1877, can be seen in Fig. 2.

### Geo-referencing maps

The aim of the georeferencing process is to correctly position the scanned map (raster image) in the chosen geographic projection. To achieve this, markers are manually placed on the scanned map to indicate that position’s location or coordinates. These markers are called ground control points (GCP).

There are various methods of transformation that the analyst can use to display the georeferenced image. These will vary in how significantly the original map’s size, shape and scale are maintained. The polynomial method forms a relationship between each of the nominated ground control points using an equation and the original map configuration. The degree of the polynomial (quadratic and cubic) affects the amount of smooth changes that can be made to the maps geometry^54^. A higher degree polynomial equation will allow for more map disfiguration, while maintaining the smooth shape of the original map. There is little further improvement for higher order polynomials beyond a cubic equation^55^.

The thin plate spline transformation is a localized method that creates envelopes around ground control points, distorting the remainder of the map around these envelopes^55^. The original shape of the map will not be maintained and some sections can become significantly warped. The notable difference between the two methods is that the polynomial transformation will aim to maintain the original scale and shape of the map, permitting the nominated ground control points to be repositioned. The thin plate spline transformation method will significantly restrict movement between the GCP points from their nominated positions, instead allowing warping of the map between designated points.

### Raster classification and generalization

Following georeferencing, the next procedure is to extract historic detail from the raster image. The raster automation process commences by first removing all unnecessary detail from the image. There is no means for the system to distinguish between any parts of the image, including what constitutes a road segment, without first completing some raster processing. Identifying which tools are effective and should be used remains the role of the user. This subsection identifies the raster editing methods used for successful map digitization in this project.

**Fig. 3:**
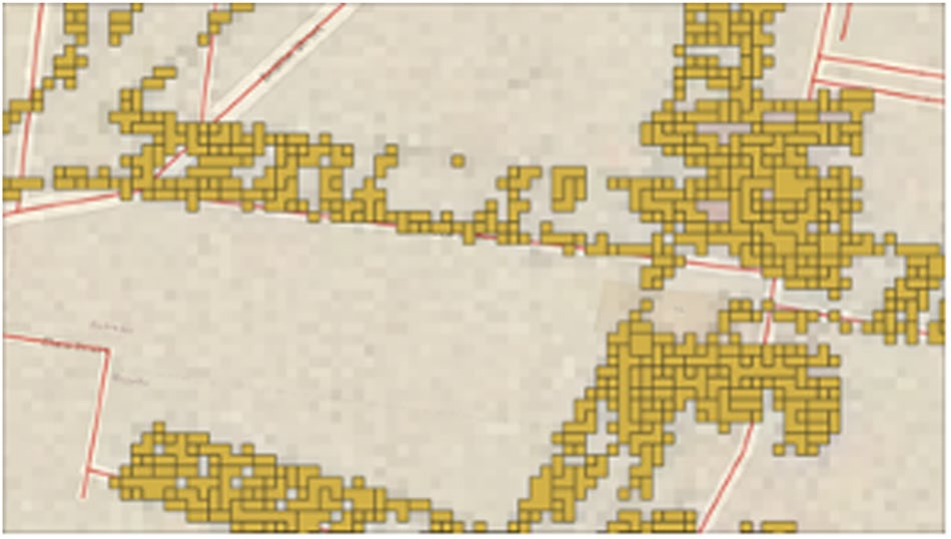
A vector layer for a road segment that has not been reclassed. Yellow polygons demonstrate the discrete and non-homogenized pixels emerged from vectorization.

The raster classes need to be edited before converting an image into a vector. A raster image is comprised of pixels, each representing different color classes, that together form the collective image. It is critical to the map digitization process that only pixels representative of road segments are maintained in the image. Further, these road segment pixels need to be homogenized into continuous pixel segments. If the image were not reclassified, each distinct class will be vectorized independently. Rather than long road sections, the vector layer would simply comprise small pixel-sized dots that cannot be easily selected, edited or utilized for further analysis. The vector structure that emerges for non-homogenized pixels can be observed in Fig. 3.

For the raster-to-vector conversion to be efficient, and for the conversion to recognize continuous segments as vectors, the color scheme for the raster image will need to be changed. Maps are scanned into a raster in an RGB color scheme, where pixels are displayed as a combination of red, green and blue color bands. From the RGB color scheme format the image is scanned in, with 255 distinct classes, the image is converted down into a binary color scheme. The scheme is structured to distinguish colors representing road segments, and other colors representing background noise.

**Fig. 4:**
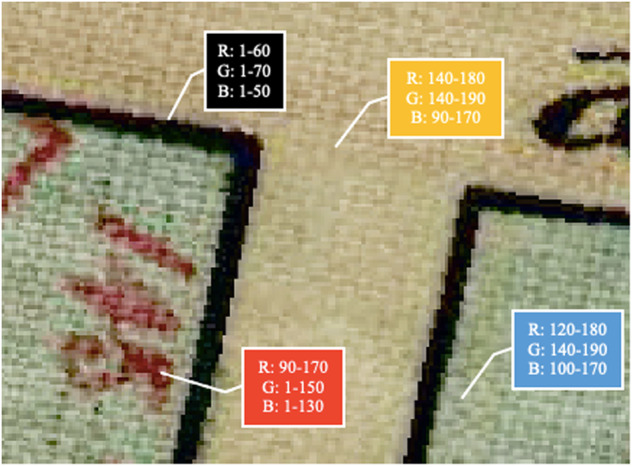
Color range for a road intersection from 1844 Woolloomooloo (inner-city eastern suburb of Sydney) map^59^.

The color classes for the scanned image will need to be divided and deconstructed in order to create a binary color scheme. An example of the class divisions is provided for a map intersection in Fig. 4. The image shows a color-enhanced intersection between two roads on a map of Woolloomooloo, dated 1844. The colors used in the image are black for road boundaries, yellow for the space between road boundaries, blue for housing or properties either side of the road, and either red or black for additional informative text. A call-out box is used to show the range of colors in each of the three color bands. For the most part, pixels representing road borders are in the range of 1-60 in the red band, while all background noise is represented outside this band. Distinguishing the image can consequentially be achieved through a cut-off in the red color band. Pixels that fall outside of this range can be considered irrelevant for road identification.

There are numerous methods that can be used to make these divisions. For Fig. 4, the color classes were identified through manual observation. The plug-in *Serval* can be used to display the RGB color palette of a raster. This is achieved through clicking pixels and displaying the color palette on screen. From observation, a color band can be selected and split into a binary selection.

However, several methods can be used to partially automate the above color identification process. It is ideal to homogenize the three color bands into one, auto-balancing the colors using the GRASS plug-in feature *i.colors.enhance*. Road features were found to typically be constructed of lower RGB values in all three bands. Comparatively, background detail was found to feature more prominently in a low color class in one band, and higher classes in the other two bands. Homogenizing the color scheme creates a meaningful cutoff that considers all color bands.

The *r.reclass* tool is then used to store all of the pixels in one of two classes, rather than the original 255. This is to create fewer vectors in the raster-to-vector conversion, making the process more efficient and connecting more vector points together during the vectorization process.

There are methods to automate the reclassification process further. A model or toolbox can be established to automate the reclassing of raster images without prompt, based on a predetermined color threshold^56^. However, this project aims to establish a method that can be considered for all map types. Considering the uniqueness of color likely to be found in maps from different times and authors, it is more accurate to observe the colors of each map and subsequently choose a color distinction. It is important to find the right class distinction. Selecting too few classes for the reclassification will remove too much information, whilst selecting too many classes will result in an overly high amount of background noise distorting results.

Two main types of errors were noticed across all the maps used in the project. Firstly, the map lines were not scanned with enough detail to display consistent and complete lines. The maps considered for this project are over a hundred years old, and understandably they would have experienced fading, damage and deterioration over time. These errors are identified in the scanning process and need to be fixed.

The intent of this raster modification is to create sharper road segments. QGIS has various in-built functions that allow for efficient raster editing. These are used to make the road segments from the scanned image as complete as possible prior to turning the map into a vector. A raster with holes, broken segments or otherwise will not transform into complete vectors, creating problems in the road identification stage later in the process. Depending on the map in consideration, raster generalization methods will have a variable rate of success.

The simple QGIS in-built program *r.grow* was used as the first means of raster editing. The feature will grow selected cells from an input criteria into their immediately neighboring cells. This was highly effective in closing gaps in road segments that were only a few pixels wide. The gap between these segments would be closed, creating longer line segments. The method was found to be effective for all maps considered for the project, and could be applied as an iterative process until road gaps were adequately closed.

An alternative to the above method is to use the SAGA plug-in program *close one cell gaps*. The program will similarly close small gaps between line segments that are slightly incomplete, however the size of the gaps that are closed does not occur to a consistent pixel size^57^. Rather, the gaps closed depend on the average characteristic of the neighbor cells, amplified by an input variable. The result is that in addition to small line gaps, the program can be more aggressively used to close the gap between entire street networks, by filling the space between two parallel road boundaries. This method is effective for closing the space efficiently in large maps, however for more intricate street networks in densely drawn suburbs, street networks tended to homogenize into one large section. This meant a loss of data about smaller streets.

### Vector transformation and vector editing

The vector layer transformed from a raster image comprises numerous lines that represent road links. The vector layer will need some processing to create a more accurate representation of the road network. The intent is to remove any features displayed in the vector layer that should not be identified by the eventual vector selection, when overlayed by the OSM layer. Various tools that were found to be effective included removing small vectors that had formed from isolated pixel clumps. Each polygon vector will contain an area that can be determined from the layer’s attribute table. All vectors with an area below a nominated size can be filtered and removed from the vector layer with relative ease. Identifying the threshold area that a polygon needs to exceed, to be maintained in the vector layer, will be unique for each map. It will depend on the average size of the features on the map. Depending on the geometries present in the map, it is possible to remove buildings, informative background texts, and small outlier pixels in this process.

Another measure of vector processing found to be effective across all maps was the *delete holes* vector geometry function. For line segments that were filled or closed in raster generalization, the function will remove all holes within a polygon vector up to a nominated area. For large vectors, the intent will be to select vectors from the base OSM layer that are completely contained within the overlayed historic GIS projection.

The alternative method of raster transformation was to use thinner lines that had only been amended on the road boundaries, not filled completely. This method was preferred for more intricately defined road networks to avoid data loss. For this method, it is convenient in the vector editing stage to extend the boundaries of the transformed polygons, using the function *buffer*. The function dissolves the space between vectors by extending the width of nominated vectors. The method is effective at automating corrective edits to sections of old maps that had not been transformed from raster-to-vector with a high degree of accuracy.

### Overlaying with OSM data

The last stage in automating the transformation of raster data into vector data involves determining which historic roads match the OSM links. The *select by location* function was utilized to determine roads from the historic street map that meet the description of an open road. To reduce confusion, cycleways and footpaths adjacent to roads were excluded from the OSM data. At intersections, there may be a slight inclusion of modern roads in the selection. To address this issue, the unintended selected features are identified and removed manually during post-processing.

In the overlaying process, the existing network (i.e., the OSM road network) is compared with historic roads. There are four possible categories for each network element in this comparison:

1. The link exists on a historic map and exists now. It’s worth noting that the link may have been constructed, demolished, and rebuilt.
2. The link exists on a historic map but not now. This could be due to (a) it was built, later demolished, or relocated, or (b) it was never built; the map was based on plans that were never realized.
3. The link does not exist on the map but exists now.
4. The link exists on neither, i.e the entire remaining space.

While this study primarily focuses on the first category, the second category was manually examined for some of the maps, and the third category is implicitly derived from the first.

## Data Records

The data records contain the Sydney road network and historic geo-referenced maps. The data consists of one shapefile (with its.dbf,.shx.cpg, and.prj extension files) and eight images (.tiff) which have been uploaded on figshare as follows:

1. **Sydney road network** (Sydney_roads.shp)^58^: This is a historic dataset for road opening dates in Sydney. The links are based on OpenStreetMap data which overlayed with the historic maps. It includes the following attributes:
  osm_id: the OpenStreetMap unique identifier for each road segment if available (integer)
  MAP001: whether the road segment exists in map Wooloomooloo, 1844. Values are ‘Open’, ‘Not Open’ or ‘Null’. Null indicates not being covered by the map.
  MAP002: whether the road segment exists in map City of Sydney, 1855. Values are ‘Open’, ‘Not Open’ or ‘Null’. Null indicates not being covered by the map.
  MAP003: whether the road segment exists in map Glebe 1888. Values are ‘Open’, ‘Not Open’, ‘Closed’ or ‘Null’. Closed indicates the road segments were open back in the date but they are closed now. Null indicates not being covered by the map.
  MAP004: whether the road segment exists in map Concord 1894. Values are ‘Open’, ‘Not Open’ or ‘Null’. Null indicates not being covered by the map.
  MAP005: whether the road segment exists in map John Sands Map of the City and Suburbs of Sydney. Published by John Sands, 1877. V Values are ‘Open’, ‘Not Open’, ‘Closed’ or ‘Null’. Closed indicates the road segments were open back in the date but they are closed now. Null indicates not being covered by the map.
  MAP006: whether the road segment exists in map John Sands Map of the City and Suburbs of Sydney. Published by John Sands, 1890. Values are ‘Open’, ‘Not Open’, ‘Closed’ or ‘Null’. Closed indicates the road segments were open back in the date but they are closed now. Null indicates not being covered by the map.
  MAP007: whether the road segment exists in map Marrickville A 1886-1888. Values are ‘Open’, ‘Not Open’ or ‘Null’. Null indicates not being covered by the map.
  MAP008: whether the road segment exists in map John Sands Map of the City and Suburbs of Sydney. Published by John Sands, 1903. Values are ‘Open’, ‘Not Open’ or ‘Null’. Null indicates not being covered by the map.
  Open2021: whether the road segment exists in OpenStreetMap data. Values are ‘Yes’ or ‘No’.
2. **Woolloomooloo 1844 historic map** (1844_Wooloomooloo_Georeferenced.tif)^59^: This is the georeferenced map of Woolloomooloo 1844 archived by City of Sydney^50^.
3. **Sydney 1855 historic map** (1855_City_of_Sydney_Georeferenced.tif)^60^: This is the georeferenced map of Sydney and suburbs 1855 archived by National Library of Australia^51^.
4. **Glebe 1888 historic map** (1888_Glebe_Georeferenced.tif)^61^: This is the georeferenced map of Glebe 1888 archived by City of Sydney^52^.
5. **Concord 1894 historic map** (1894_Concord_Georeferenced.tif)^62^: This is the georeferenced map of Concord 1894 archived by Dictionary of Sydney^53^.
6. **City and Suburbs of Sydney 1877 historic map** (1877_Sydney_Georeferenced.tif)^63^: This is the georeferenced map of City and Suburbs of Sydney 1877 archived by National Library of Australia^47^.
7. **City and Suburbs of Sydney 1890 historic map** (1890_Sydney_Georeferenced.tif)^64^: This is the georeferenced map of City and Suburbs of Sydney 1890 archived by State Library of NSW,^49^.
8. **Marrickville 1886-1888 historic map** (1888_Marrickville_Georeferenced.tif)^65^: This is the georeferenced map of Marrickville 1886-1888 archived by Dictionary of Sydney^53^.
9. **City and Suburbs of Sydney 1903 historic map** (1903_Sydney_Georeferenced.tif)^66^: This is the georeferenced map of City and Suburbs of Sydney 1903 archived by National Library of Australia^48^.

## Technical Validation

**Fig. 5:**
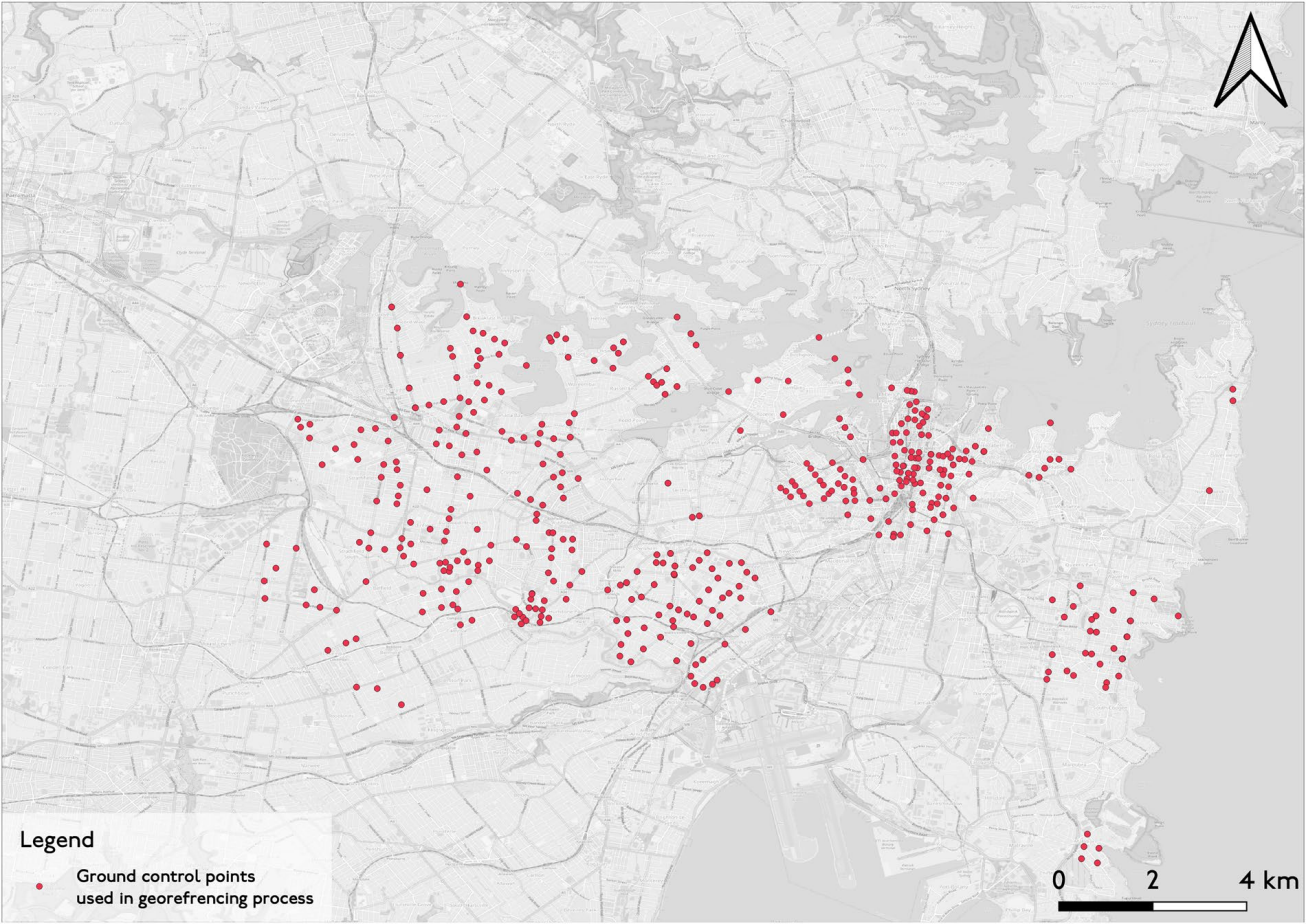
Ground control points used in georeferencing the historic maps.

The georeferencing requirements for this study focus on accurately positioning large maps. A visual comparison was completed for the John Sands 1903 map. With the establishment of ground control points (GCPs), as illustrated in Fig. 5, the georeference was completed using two transformation methods: polynomial 3 and thin plate spline. These GCPs are manually pinpointed across the maps’ periphery and center. The thin plate spline method becomes increasingly effective when compared to the polynomial transformation method, as more ground control points are nominated. This is a result of the ability to remove local errors in the mapping^67^. However, this depends on the evenness of the ground control point distribution. A neglected area will degrade significantly in either method of transformation^68^.

**Fig. 6:**
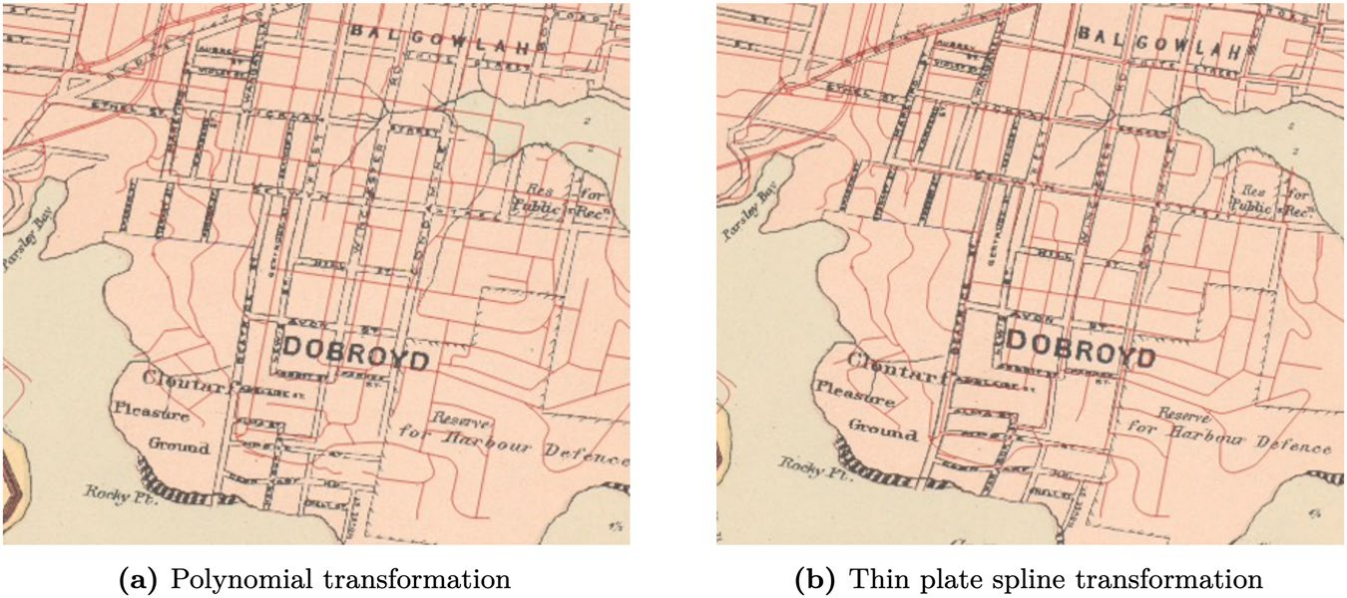
Map of the suburb of Manly. Georeferenced with two different methods: polynomial and thin plate spline. OpenStreetMap (present day) shown in red.

The difference in the two methods is quickly apparent from a comparison. Figure 6 shows the final cubic polynomial and thin plate spline georeference transformations for the suburb of Manly, a small selection from the whole map. Both maps are overlayed by the present OpenStreetMap vector layer, and both layers feature identical ground control points. The thin plate spline transformation layer is distorted as a result of the transformation process, however the road sections have far closer alignment to the OSM layer. The cubic polynomial transformation is shifted below the OSM layer, a result of trying to retain the shape of the scanned map. Whilst neither map is perfect, and there will be some rectification required for both maps, the noticeably closer match between the OSM layer and the thin plate spline transformation will return far fewer errors that need to be amended in the remaining automation steps.

**Fig. 7:**
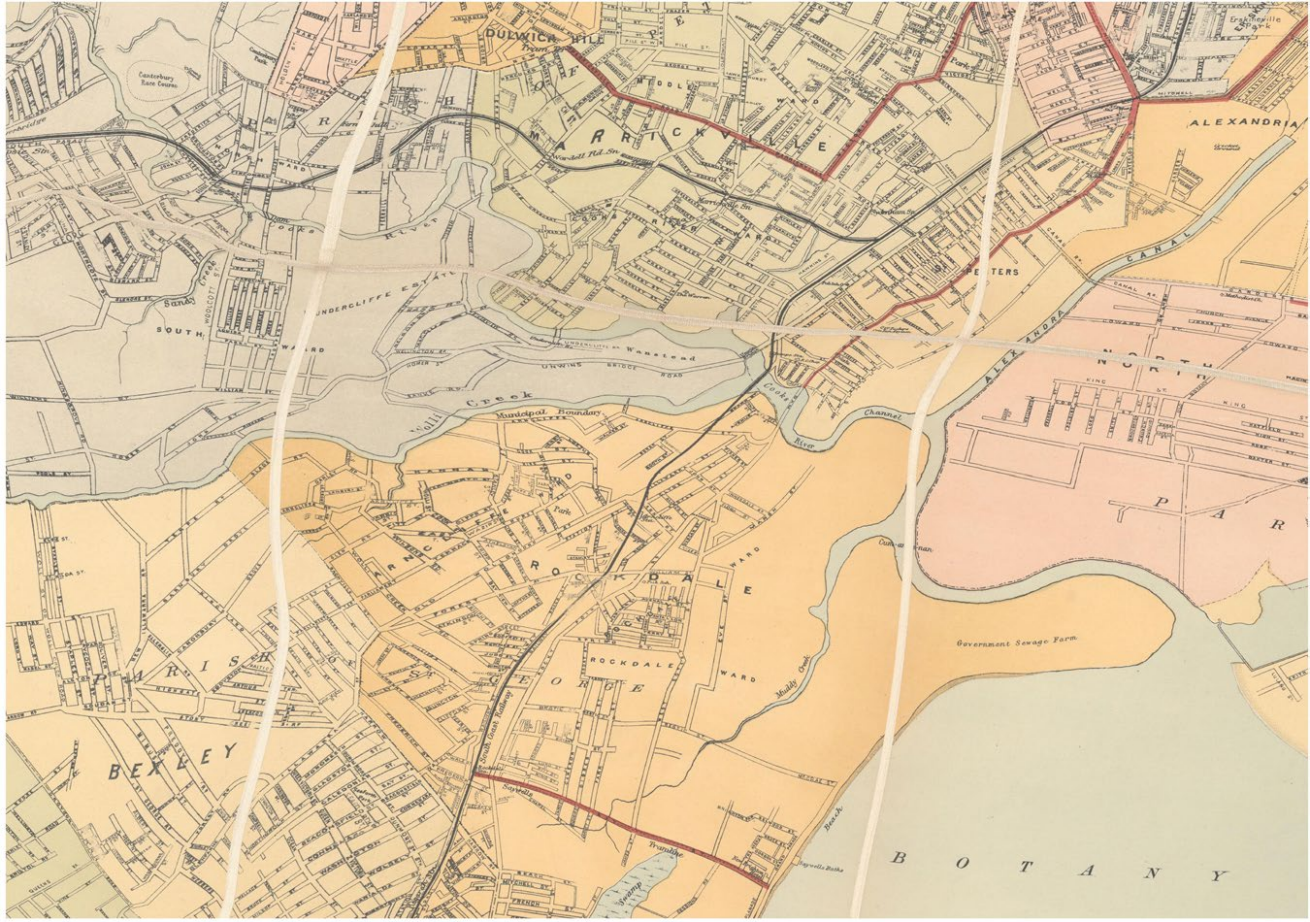
Map of Botany Bay and neighboring suburbs 1903^66^, showing distorted grid lines.

By significantly georeferencing larger maps using the thin plane spline method, the original integrity of the map will be lost. Whilst previous examples highlighted the benefit of this distortion for vector matching on a localized scale, the end result of substantial thin plate spline transformations can be significantly deconstructive to the original state of the map. In Fig. 7, a thin plate spline transformation of the John Sands map published in 1903, the global distortion caused by the georeference of the map can be clearly observed. In the images, multiple suburbs are observed to the west of Botany Bay. Thick, white grid lines in the original map represent planar folds from when the map has been folded over time, presumably with the intent to fold along these lines and minimize damage to the remaining image. These grid lines were rigidly drawn with sharp edges. However, the grid lines have been curved and distorted significantly through the georeference transformation, in order to correctly position the map. It highlights errors in the construction of the original map, where suburbs were not positioned in the correct size and scale, rather than errors in the georeferencing process. The aim of georeferencing is to correctly position the map and this can be at the expense of the historic maps original shape and form. Consequentially, the additional distortion caused by the thin plate spline method should not cause concern when completing the georeferencing transformation.

To validate the applied georeferencing method, a comparison of two algorithms has been conducted on a relatively small map of the suburb of Marrickville published in 1888^53^. The two transformation methods were chosen consistently with the methods used for larger maps: polynomial 2 and thin plate spline. Table 1 summarizes the georeferencing methods. Both methods have been compared, alongside a comparison of the amount of ground control points used for the transformation. A small selection of 6 ground control points quickly positioned one key intersection of the map from a 3x2 grid. Selecting 45 ground control points covered all the main intersections in the map network.

**Table 1:** Georeferencing methods.

Transformation	Points (Total 1357)	Mean Error (m)
Polynomial 2	6	0.00
Thin Plate Spline	6	0.00
Polynomial 2	45	3.39
Thin Plate Spline	45	2.3 × 10^-11^

In Table 1, mean error is in reference to the map distortion in x and y co-ordinates of GCP points after selecting by the map canvas. Mean error may be influenced by a poorly selected ground control point, however it may also be influenced by poor geo-spatial properties of the original map, such as size or scale. As a result, generating a mean error may not necessarily be detrimental to the geo-referencing accuracy, if it fixes errors in the historic map. Closely aligning the historic and base maps is the intent of the geo-referencing process. A visual comparison of the maps shows there to be little difference between the four maps on first analysis.

## Usage Notes

**Fig. 8:**
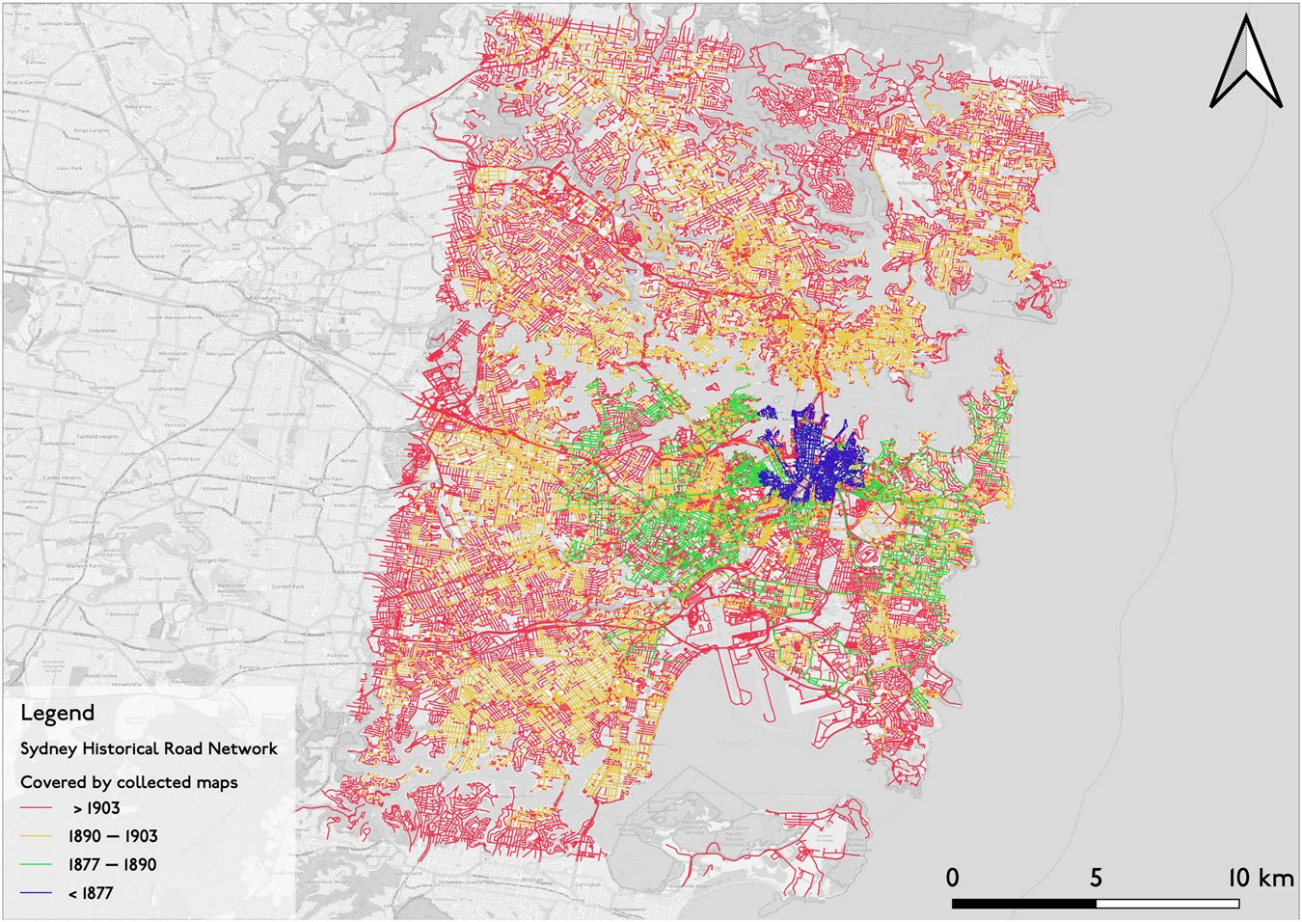
Sydney historical road network by year. Basemap: OpenStreetMap.

The primary application of the methods detailed in the previous sections was completed on the John Sands map series spanning from 1877 to 1903. A partially automated process for map digitization was applied to each map in the series. A vector layer (i.e., shapefile) similar to the OSM road network is labeled for each map, as described in the Data Records section. Using the defined attributes, one can extract the road network of each map by deconstructing the shapefile into different time intervals representing the open status of the road network in Sydney as of 1877, 1890, and 1903, and beyond. For example, Fig. 8 visualizes the road network over the time period covered by the maps.

The history of demographics in cities, for example, urban migration, can be evaluated against the roads that served these movements. The evolution of transit networks and their impacts on population distribution have been studied in the literature^24,69–72^. With the current dataset one can conduct similar analyses and investigate the interaction between land use and the road network.

The reliability of the current dataset depends on the accuracy and level of detail in these maps. This dataset classifies streets based on their presence in historical maps as ‘Open,’ ‘Not Open,’ or ‘Null’ (i.e., whether a street was or wasn’t present on a historic map), and it does not provide absolute certainty regarding the physical existence of these streets in those times. This limitation is acknowledged to ensure that users understand the technical meanings of the classifications and their constraints. While this dataset serves as a valuable resource for historical reference, it may require additional primary data verification for absolute confirmation of the historical existence of streets.

## Data Availability

No custom code was used to generate or process the data described in this article.
